# A Three-Day Intervention With Granola Containing Cereal Beta-Glucan Improves Glycemic Response and Changes the Gut Microbiota in Healthy Individuals: A Crossover Study

**DOI:** 10.3389/fnut.2022.796362

**Published:** 2022-04-28

**Authors:** Vibeke H. Telle-Hansen, Line Gaundal, Benedicte Høgvard, Stine M. Ulven, Kirsten B. Holven, Marte G. Byfuglien, Ingrid Måge, Svein Halvor Knutsen, Simon Ballance, Anne Rieder, Ida Rud, Mari C. W. Myhrstad

**Affiliations:** ^1^Department of Nursing and Health Promotion, Faculty of Health Sciences, Oslo Metropolitan University, Oslo, Norway; ^2^Department of Nutrition, Faculty of Medicine, Institute of Basic Medical Sciences, University of Oslo, Oslo, Norway; ^3^The Norwegian National Advisory Unit on Familial Hypercholesterolemia, Department of Endocrinology, Morbid Obesity and Preventive Medicine, Oslo University Hospital Rikshospitalet, Oslo, Norway; ^4^Mills AS, Oslo, Norway; ^5^Nofima AS (Norwegian Institute of Food, Fisheries and Aquaculture Research), Ås, Norway

**Keywords:** soluble fiber, beta-glucan, fiber, humans, crossover study, dietary intervention, glycemic response, gut microbiota

## Abstract

Intake of soluble fibers including beta-glucan, is known to improve post-prandial glycemic response. The mechanisms have been attributed to the viscous gel forming in the stomach and small intestine, giving a longer absorption time. However, recent evidence suggests a link between intake of beta-glucan and improved glycemic regulation at subsequent meals through the gut microbiota. We investigated the short-term effect of granola with different amounts of cereal beta-glucan on glycemic response and gut microbiota. After a two-week run-in period (baseline), fourteen healthy, normal weight adults completed a dose-response dietary crossover study. Different amounts of cereal beta-glucan (low: 0.8 g, medium: 3.2 g and high: 6.6 g) were provided in granola and eaten with 200 ml low-fat milk as an evening meal for three consecutive days. Blood glucose and insulin were measured fasted and after an oral glucose tolerance test (OGTT) the following day, in addition to peptide YY (PYY) and glucagon-like peptide (GLP-2), fasting short chain fatty acids (SCFA) in blood, breath H_2_, and gut microbiota in feces. Only the intervention with medium amounts of beta-glucan decreased blood glucose and insulin during OGTT compared to baseline. Fasting PYY increased with both medium and high beta-glucan meal compared to the low beta-glucan meal. The microbiota and SCFAs changed after all three interventions compared to baseline, where acetate and butyrate increased, while propionate was unchanged. Highest positive effect size after intake of beta-glucan was found with *Haemophilus*, followed by *Veillonella* and *Sutterella*. Furthermore, we found several correlations between different bacterial taxa and markers of glycemic response. In summary, intake of granola containing 3.2 g cereal beta-glucan as an evening meal for three consecutive days reduced the glycemic response after an OGTT 0-180 min and changed gut microbiota composition. Since we cannot rule out that other fiber types have contributed to the effect, more studies are needed to further explore the effect of cereal beta-glucan on glycemic regulation.

**Clinical Trial Registration:**

[www.clinicaltrials.gov], identifier [NCT03293693].

## Introduction

Consumption of cereal fiber promotes positive health effects, including reduced risk of type 2 diabetes (T2D) (1–3). Foods containing sufficient amounts of beta-glucan, a soluble fiber found primarily in barley and oat grains, have been shown to reduce post-prandial glycemic response in humans (4). The beneficial effect of cereal beta-glucan has been explained by the formation of a viscous solution in the small intestine and/or increased barrier function of the mucus layer (5), delaying the absorption of glucose and reducing the post-prandial glycemic response (6, 7). Delayed absorption is also the established mechanistic explanation of the reduction in blood cholesterol and re-absorption of bile acids (8–14) observed after fiber and beta-glucan intake, and thereby reducing the risk of cardiovascular diseases (CVD) (15). Beta-glucans’ ability to form viscous solutions is highly dependent on their molecular weight, solubility and concentration. The effect of cereal beta-glucan on post-prandial glycemia is approved as a health claim by the European Food Safety Authority (EFSA) for products containing 4 g beta-glucan per 30 g available carbohydrates (16). Even so, the amount of beta-glucan giving a reduction in post-prandial glycemic response has been discussed (17). Furthermore, while the effect of cereal beta-glucan on post-prandial glycemic response is well established, its potential to modulate glycemic response at subsequent meals has recently been investigated. Studies have shown a beneficial effect of barley, which is high in beta-glucan, on blood glucose regulation also after a subsequent meal and overnight fast (18–20), indicating other mechanisms than the formation of a viscous gel and delayed post-prandial glucose uptake to be involved. However, information about the beta-glucan dose and characteristics is often lacking and the beta-glucan dose necessary for such an effect is not known.

Changes in dietary fiber intake may also affect human health indirectly through changes in the gut microbiota. The gut microbiota and its host are in a symbiotic relationship by a joint utilization of consumed nutrients, and the fermentation products produced by the human gut microbiota are hypothesized to play a major role in host energy and substrate metabolism (21). Short chain fatty acids (SCFA) are fermentation products that are increased after fiber intake and suggested to be involved in metabolic regulation in the host, including glycemic regulation (22, 23). A positive correlation between colonic fermentation of fiber and improved glucose tolerance has been proposed as a plausible mechanism (24). SCFA have also been implicated in the regulation of secretion of several gut hormones, including glucagon-like peptide 1 (GLP-1) and peptide YY (PYY), shown to regulate energy homeostasis and glucose metabolism (25, 26). GLP-1 is well known to affect glycemic regulation, and studies suggest that also GLP-2 exerts beneficial effects on glucose metabolism (27, 28). Changes in metabolic regulation due to beta-glucan intake, have been associated with increased levels of SCFA, particularly butyrate, and higher levels of PYY and GLP-1 in mice (29). However, the potential involvement of gut microbiota in glycemic regulation needs further investigation.

The aim of the present study was therefore to investigate the effect of different amounts of beta-glucan from oat and barley after a short-term intervention on glycemic response in healthy subjects and relate the effects on glycemic response to changes in gut microbiota.

## Materials and Methods

### Study Design and Subjects

**FIGURE 1:**
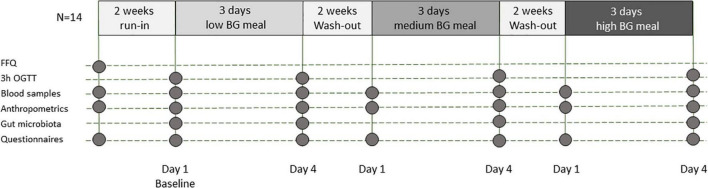
Study design. BG: beta-glucan, FFQ: food frequency questionnaire; h: hours; N: number of participants; OGTT: oral glucose tolerance test.

**FIGURE 2:**
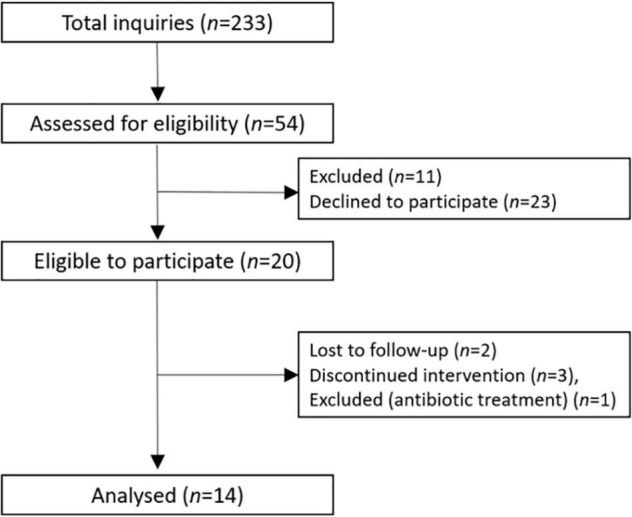
Flow-chart of the participants.

Healthy volunteers were recruited to participate in this dose-response, single blind, controlled study, lasting for nine weeks with three test weeks (Figure 1). The study was performed at Oslo Metropolitan University (OsloMet) between September 2016 and March 2017. Twenty participants met for the first visit whereas 14 completed the study and are included in the statistical analyses as outlined in Figure 2. Students and employees at OsloMet were recruited to the study through presentations in classes, e-mails, and via social media. The subjects met for six visits during the study. After two weeks run in, participants received test meals with low, medium and high amounts of beta-glucan in the first, second, and third test week, respectively. The test weeks were followed by two weeks wash out periods. A wash out period of two weeks was chosen to ensure no carryover effect between test weeks. Prior to each visit, the subjects were asked to fast for 12 h and refrain from alcohol and excessive physical activity the day prior to the visits. A standard oral glucose tolerance test (OGTT) was performed after run-in, at baseline and after each test week with low, medium and high beta-glucan doses, altogether four times. Clinical assessment and blood samples were collected at every visit, as well as urine and feces samples, and breath H_2_. A case report form was used to assess compliance to the regimen, changes in diet or physical activity level, overall health status, and if the participants reported adverse effects. A food frequency questionnaire (FFQ) to map the participants’ habitual diet in the past 12 months was completed before study start.

Exclusion criteria were fasting blood glucose ≥ 6.1 mmol/L, CRP > 10 mg/L and BMI < 18.5 and > 27 kg/m^2^ and planned weight reduction and/or 5% weight change previous three months, use of antibiotics the last three months and during the study period, smoking, and hormonal treatment (except oral contraception), or blood donation the last two months and during the study. In addition, pregnant or lactating women, and people with food allergies or intolerances, chronic metabolic diseases and inflammatory bowel diseases were excluded. The subjects had to be willing to limit their consumption of dietary products rich in beta-glucan (oat and barley-based products) and whole grain products during the whole study period, including the run-in and wash-out periods. The subjects had to stop using any dietary supplements and probiotic products (lactic acid bacteria) four weeks prior to, and during the study. The subjects were asked not to change their physical activity level and dietary habits (except from the restrictions) during the study.

The study was conducted according to the guidelines in the Declaration of Helsinki and approved by the Regional Committees for Medical and Health Research Ethics (REK 2016/648) and registered at clinicaltrials.gov (NCT03293693). Written informed consent was obtained from all participants.

### Test Meals

The test meals consisted of 100 g cereal, based on the commercially available product Vita hjertego’ Granola provided by Mills AS. The granola meals contained low, medium, or high amounts of beta-glucan and were consumed with 200 ml low-fat milk as an evening meal for three consecutive days. The medium beta-glucan test meal was identical to the commercial product and consisted of the following cereals: rolled oats (28%), rolled rye (13%), barley flakes (11%), oat bran (5%), puffed spelt (5%), and rye flakes (8%), in addition to oat fiber (1.6%), barley fiber (1.9%), and inulin (6.7%). In the low beta-glucan test meal, the beta-glucan containing cereal and fiber ingredients (all barley and oat-based ingredients) were replaced with rolled wheat and buckwheat flakes and oat and barley fiber were exchanged with inulin by the manufacturer. The test meal with high beta-glucan content was composed of 60 g commercial granola (medium beta-glucan test meal) and 40 g of an extruded oat product with very high beta-glucan content and high beta-glucan molecular weight available in pharmacies as functional food (“Betavivo havrehjerter,” Oatwell). The test meals were packaged into 100 g servings in neutral package with similar appearance at OsloMet and given to the participants at each visit.

Energy and macronutrients in the test meals were determined by a routine food analysis laboratory (Eurofins Food & Feed Testing Norway AS) with standard analysis. To estimate the content of soluble and insoluble fiber and differentiate between fiber types, soluble and insoluble non-starch polysaccharides (NSP) were determined as alditol acetates of their constituent sugars (30). Arabinoxylan content was estimated from alditol acetates as the sum of arabinose and xylose, while cellulose content was estimated as the total glucose minus beta-glucan. Beta-glucan weight average molecular weight of test meals was determined by HPSEC with a multi-angle light scattering detector, a viscometer and a refractive index detector as (SEC MALLS) previously described (31). Prior to molecular weight analysis, beta-glucan was extracted from the test meals by using an enzymatic extraction (thermostable alpha-amylase and xylanase) after an inactivation of endogenous enzymes by boiling in 50% ethanol (31). The study products had similar energy contents and only small differences in fat (< 5 g/100 g for all), protein (8–13 g/100 g), available starch (46–53 g/100 g) and sugar content (8.7–14 g/100 g) (Table 1). Dietary fiber content in the test products varied from 8.7 g/100 g (low beta-glucan) to 17.9 g/100 g (high beta-glucan). However, dietary fiber determined by AOAC 985.29 does not include short chain (soluble in aqueous ethanol) soluble dietary fibers such as inulin. Inulin was quantified in all test products and varied from 7.6 g/100 g (low beta-glucan) to 4.1 g/100 g (high beta-glucan). Hence, total dietary fiber was calculated as the sum of dietary fiber and inulin and ranged from 16.3 g/100 g (low beta-glucan) to 22 g/100 g (high beta-glucan). The individual composition of different fiber types in the test products, including beta-glucan amounts, are shown in Table 1.

**TABLE 1:** Macronutrient and chemical composition and properties of dietary fibers in the test meals with and without *in vitro* digestion^a^.

	Low beta-glucan	Medium beta-glucan	High beta-glucan
Energy (kcal/serving)^c^	355	347	361
Fat^c^	2.3	3.8	4.4
Protein^c^	8.3	9.8	13.2
Available starch^c^	53 ± 2	51 ± 3	46 ± 2
Sugars^c^	14 ± 0.1	8.7 ± 0.1	9.8 ± 0.3
Dietary fiber^c^	8.7	12.2	17.9
Inulin^c^	7.6 ± 0.3	6.2 ± 0.1	4.1 ± 0.1
Total dietary fiber^b,c^	16.3	18.4	22.0
Beta-glucan^d^	0.8	3.2	6.6
Resistant starch^d^	0.4	0.5	0.2
Arabinoxylan^d^	4.1	4.6	4.7
Cellulose^d^	1.1	2.6	2.9 ± 0.5
Other NSP^d^	0.9	1.1	1.1
SUM insoluble NSP^d^	4.6 ± 0.2	5.3 ± 0.2	6.4 ± 0.2
SUM soluble NSP^d^	2.2 ± 0.3	6.1 ± 0.2	8.8 ± 0.2
Total soluble fiber (soluble NSP + inulin)^d^	9.8 ± 0.3	12.3 ± 0.2	12.9 ± 0.2
Beta-glucan M_*w*_ (SEC MALLS) (kDa)^d^		1692	1859
*In vitro digestion (supernatant)^d^*			
Zero shear viscosity (mPas)	1.1	3.2	114 ± 2
Beta-glucan solubilization (% of total beta-glucan)	17.0	28 ± 3.5	40 ± 5
*In vitro digestion (pellet)^d^*			
Insoluble beta-glucan, cellulose and resistant starch	14.3 ± 1.1	16.8 ± 1.1	13.3 ± 0.8
Insoluble arabinoxylan	3.7 ± 0.1	3.3 ± 0.3	3.7 ± 0.1
Sum insoluble fiber	18.2 ± 1.2	20.4 ± 1.6	17.4 ± 0.9

*^a^All results are in g/100g test meal if not otherwise stated. Serving size was 100g. Average values of two parallels are presented with SD larger than 0.1. SD smaller than 0.1 are not included in the table.*

*^b^Since dietary fiber analysis according to AOAC 985.29 does not include inulin, total dietary fiber is given as the sum of dietary fiber (AOAC 985.29) and inulin (AOAC 999.03).*

*^c^Analyses performed at Eurofins.*

*^d^Analyses performed at Nofima. kDa: kilodalton, NSP: Non-starch polysaccharides, M_w_: weight average molecular weight, SEC MALLS: Size exclusion chromatography with multi angle laser light scattering detection.*

The test meals provided 0.8, 3.2 and 6.6 g of beta-glucan per day. The medium dose (3.2 g per day) corresponded to the requirements for the EFSA health claim on reduction of blood cholesterol of 1g per serving and at least 3 g per day (32), while the highest dose nearly fulfilled the criteria of the EFSA health claim for reduction of post-prandial glycemic response as it contained 3.5 g beta-glucan per 30 g available carbohydrate (16) (Table 1). Apart from inulin and beta-glucan, the other fiber types such as cellulose (1.1–2.9 g/100 g), arabinoxylan (4.1–4.7 g/100 g) and resistant starch (0.2–0.5 g/100 g), did not vary much between test products. The low beta-glucan test meal contained less fiber, both soluble and insoluble than the other two, while medium and high beta-glucan test meals had similar amounts of soluble fiber with 12.3 and 12.9 g/100 g, respectively. The medium and high beta-glucan test meals had beta-glucan with a high weight average molecular weight of 1690 kDa (medium beta-glucan) and 1860 kDa (high beta-glucan) (measured by SEC MALLS). The beta-glucan in the low beta-glucan test meal originated from wheat and was not soluble enough to determine molecular weight (Table 1).

### Physicochemical Characteristics of the Test Meals

To determine the physicochemical characteristics of beta-glucan and other fibers in the test meals under physiological conditions, all test meals were subjected to an *in vitro* digestion procedure based on the Infogest protocol (33). Viscosity, beta-glucan molecular weight (with SEC-post column calcofluor) and beta-glucan solubility were determined from the *in vitro* digested extracts as previously described (34). The pellets from *in vitro* digestion were analyzed for undigested insoluble polysaccharides as the alditol acetates of their sugar constituents generated during acid hydrolysis analyzed by GC-FID (30).

### Oral Glucose Tolerance Test

A standard OGTT was performed at baseline and after each test week, altogether four times. The participants met after an overnight fast (≥ 12 h) for an OGTT and were instructed to refrain from alcohol consumption and excessive physical activity the day before. Eighty-two gram of glucose [D(+)-Glucose monohydrate], equal to 75 g glucose, were dissolved in 100 ml water and stored in a refrigerator for maximum two weeks. The participants were instructed to consume the OGTT within 10 min and to remain seated between the measurements following the OGTT. Finger-prick capillary blood samples for glucose measurements were taken before and 15, 30, 60, 90, 120, 150, and 180 min after the OGTT. Venous blood samples for other biochemical measurements were taken before and 30, 60, and 120 min after the OGTT.

### Clinical and Biochemical Measurements

Blood glucose concentration was measured at OsloMet using HemoCue Glucose 201 Analyzer and Microcuvettes. The Microcuvettes were stored in a refrigerator (4°C) and taken out in room temperature 30 min prior to blood sampling. Insulin and hsCRP were measured in serum before, at 30, 60 and 120 min after the OGTT. Triglycerides and total cholesterol were measured in serum fasted at every visit. Serum was obtained from 8.5 ml serum gel tubes and turned 6-10 times before spin down after 30 min (1300 – 1500 g, 15 min), and kept in a refrigerator (4°C) before it was sent to a routine laboratory (Fürst Medical Laboratory, Oslo, Norway) within 24 h.

Peptide YY (PYY) and glucagon-like peptide 2 (GLP-2) were measured in plasma before and 30, 60, and 120 min after the OGTT. Plasma was obtained from EDTA-tubes, immediately placed on ice and centrifuged within 10 min (1500 g, 4°C, 10 min). EDTA-plasma were stored at −80°C and shipped to a commercial laboratory for analysis (Vitas Analytical Service, Oslo, Norway).

### Short Chain Fatty Acids Analysis

SCFA was measured fasted in EDTA plasma. EDTA plasma was obtained fasting at baseline and after each test week. EDTA-tubes were immediately placed on ice and centrifuged within 10 min (1500 g, 4°C, 10 min) and EDTA plasma was stored at −80°C until shipped to a commercial laboratory for analysis (Vitas Analytical Service, Oslo, Norway).

### Anthropometry

Body weight and composition was measured after an overnight fast at each visit using a Tanita scale (BC-418 Segmental Body Composition Analyzer). Any metal (i.e., watch, jewelry, belt etc.), shoes and socks were removed before the measurement. One kg was subtracted from the body weight, compensating for clothing. Height was measured by a wall-mounted stadiometer.

### Breath H_2_

As an indicator of colonic fermentation, expiratory hydrogen (H_2_) was measured using a Gastro^+^™ gastrolyzer (Bedfont Gastrolyzer). The subjects were asked to inhale and hold their breath for 15 s before exhaling into a plastic tube in an evenly pace until all air was completely exhaled. Breath H_2_ was measured fasted and 15, 30, 60, 90, 120, 150 and 180 min after the OGTT. Values of expiratory H_2_ are expressed as parts per million (ppm).

### Microbiota Analysis

The subjects were asked to deliver fecal samples at every visit. At each test week, fecal samples were collected before each test meal and from the first stool after consumption of the last evening test meal. The subjects received a kidney bowl and were asked to sample the feces on three different places of the stool. The participants were asked to store the samples at 4 degrees until delivery at OsloMet at the next visit. Fecal samples were stored at −20°C at OsloMet before they were shipped to Nofima for microbiota analyses. Two fecal samples were missing, giving a total of 82 fecal samples analyzed for microbiota.

Bacterial DNA was extracted from fecal content (approximately 100 mg) of 82 samples by mechanical and chemical lysis using the PowerLyzer™ PowerSoil^®^ kit (MoBio Laboratories), following the manufacture’s protocol. The mechanical lysis step with bead beating was done twice using the FastPrep^®^-24 homogenizer (MP Biomedicals) for 40 s at 6m/s.

The microbiota analysis was performed by high throughput sequencing following in-house protocol (35), which is presented in detail in supplementary methods of Caporaso et al. (36). The method involves paired end sequencing (2 × 150bp) of the variable region 4 of the bacterial 16S rRNA gene. The sequencing was done on a MiSeq (Illumina) at Nofima using pooled polymerase chain reaction (PCR) samples, which were based on triplicate PCRs per DNA sample using sample-specific barcoded forward primers. PhiX Control v3 was included and accounted for 10% of the reads. The MiSeq Control Software (MCS) version used was RTA 1.18.54.

Data processing of the sequencing reads was performed using the open-source bioinformatics pipeline Quantitative Insight Into Microbial Ecology (QIIME) v.1.8 (37). Briefly, the forward and reverse reads were joined, and barcodes failed to assemble were removed, resulting in 11.5 million reads. The sequences were demultiplexed into representative sample taqs and quality filtered allowing zero barcode errors and a quality score of 30 (Q30). Reads were assigned to their respective bacterial taxonomy (Operational Taxonomic Unit: OTU) by clustering them against the Greengenes reference sequence collection (gg_13_8) using a 97% similarity threshold. Reads that did not hit a sequence in the reference sequence collection were clustered *de novo*. Chimeric sequences were removed using ChimeraSlayer, and all OTUs that are observed fewer than 2 times were discarded. This resulted in an OTU table containing 19,741 different OTUs from a total of 7.8 million OTU counts. The OTU table was used for alpha diversity rarefaction analysis using equal number of sequences across samples (i.e., 60,000 sequences per sample). Taxonomic summary tables at phylum and genus levels were constructed from the OTU table. The data were transformed by centered log2 ratios, to stabilize the variation and remove dependencies between abundance variables. Phyla or genera that were present in less than 50% of the subjects were combined into one group (called “rare”), as it is not possible to make statistical inference on individual rare bacteria groups.

### Statistical Analysis

The primary outcome measures as described in Clinical Trials are postprandial blood glucose and insulin response after an OGTT (glycemic response) and the statistical power was estimated for the glucose response. Secondary endpoints were related to gut microbiota, hunger and satiety. Sample size was calculated based on a previous study on observations of an evening meal consisting of barley and effects in blood glucose incremental Area Under the Curve (iAUC) 0–120 min (20). We calculated that the number of participants had to be between 13 and 17. This was based on a strength of 80% and accepted level of type 1 error of 5%. We therefore wanted to include 20 people in this study to take into account a 20% drop-out. Due to sample size, data were analyzed with non-parametric tests and are presented as median and 25^th^–75^th^ percentiles. Friedman’s ANOVA and Wilcoxon Signed Rank Test was used to assess differences between interventions with low, medium, and high beta-glucan and baseline measurements. Baseline measurements was obtained after run-in and before the intake of the low dose beta-glucan. The iAUC was calculated for each participant by subtracting the fasting value (0 min) at each visit from the corresponding values after the OGTT, and thereafter using the trapezoidal rule [A = (y1 + y2) * (x2 – x1)/2]. Homeostasis model assessment of insulin resistance (HOMA-IR) (38) and Matsuda Index (39) were calculated for each participant to assess insulin resistance and insulin sensitivity, respectively. *P* < 0.05 was regarded as statistically significant. For one participant, there was one missing value (insulin at 60 min after OGTT at visit 5). As a replacement, we used the mean value for all the other participants at the same time point. All statistical analyses were performed in IBM SPSS statistic.

The variation in microbiota at phylum and genus level were decomposed by analysis of variance simultaneous component analysis (ASCA) (40, 41). The ASCA model contained a Subject effect, accounting for the between subject variation, and an intervention-specific visit effect. *Post hoc* comparisons between factor levels of the intervention design were performed using partial least squares discriminant analysis (PLS-DA) after removing the between-subjects variation (42). Bacteria that discriminate the test meal from its baseline level were identified by variable importance in prediction (VIP). A cut-off of 1.0 was used for VIP. Effect sizes were calculated as difference between means after test meal compared to baseline. The microbial diversity, represented by the metrics Observed OTUs, Phylogenetic Distance (PD) whole tree and Chao1, was analyzed using a Mixed-Effects Model in Minitab ^®^19.2 where visit was defined as fixed effect and subject as random.

## Results

### Baseline Characteristics

Of the twenty volunteers that were randomized, six subjects were lost during follow-up (Figure 2). The fourteen participants that completed the study, 12 women and 2 men, were healthy, in the min-max age span between 20 and 46 years and with a median body mass index (BMI) of 22.2 kg/m^2^ (Table 2). The background diet (measured before run-in) of the participants consisted of a total fat intake in the upper range of the recommendation with a median intake of 36.5 E%. Saturated fatty acids (SFA) were higher than recommended (12.3 E%), while monounsaturated fatty acids (MUFA) and polyunsaturated fatty acids (PUFA) were in the lower range (13.6 E% and 6.1 E%, respectively). Intake of proteins and carbohydrates were 17.6 E% and 42.9 E%, respectively. Total fiber intake was high with a median intake of 35.8 g per day for the total group (Table 3).

**TABLE 2:** Baseline characteristics of participants.

Gender *n* (F/M)	12/2
Age (years)	28.0 (24.0 – 38.3)
BMI (kg/m^2^)	22.2 (20.8 – 24.2)
Fasting glucose (mmol/L)	5.1 (4.8 – 5.7)
Fasting insulin (pmol/L)	65.5 (58.8 – 83.8)
Fasting total cholesterol (mmol/L)	4.4 (3.8 – 4.5)
Fasting triglyceride (mmol/L)	0.74 (0.65 – 0.87)
Fasting hsCRP (mg/L)	0.5 (0.3 – 1.0)

*Data is given as median (25^th^**–**75^th^ percentiles) except for gender. BMI: Body mass index, hsCRP: high sensitivity C-reactive protein.*

**TABLE 3:** Background diet.

	Median	25^th^ – 75^th^
Energy (kJ)	9272.0	8031.5-10146.0
Protein (E%)	17.6	14.7-18.7
Fat (E%)	36.5	28.7-39.8
SFA (E%)	12.3	10.9-14.3
MUFA (E%)	13.6	10.0-15.2
PUFA (E%)	6.1	5.3-6.9
Carbohydrates (E%)	42.9	37.2-49.0
Fiber (g/day)	35.8	27.7-43.3

*Macronutrient intake assessed from FFQ.*

*Data is given as median with 25^th^–75^th^ percentiles.*

*FFQ: food frequency questionnaire, kJ: kilojoule, E%: percentage of total energy intake, g/d: gram/day, SFA: saturated fatty acids, PUFA: Monounsaturated fatty acids, PUFA: Polyunsaturated fatty acids.*

### Glycemic Regulation After Intake of Granola With Cereal Beta-Glucan

**FIGURE 3:**
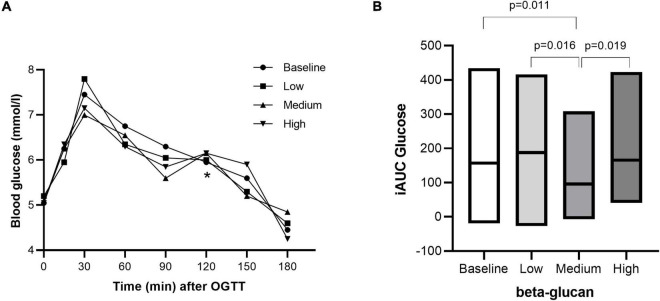
Postprandial blood glucose response after intake of granola with cereal beta-glucan. The postprandial glucose response from 0–180 min **(A)** and the incremental area under the curve **(B)** after an OGTT at baseline and after consuming low, medium and high amount of beta-glucan for three days. Data is given as median (25^th^–75^th^). Significant differences are calculated with Friedman’s Anova followed by Wilcoxon signed rank test. Significant differences are indicated with * **(A)** or with a *p-value* **(B)**.

**FIGURE 4:**
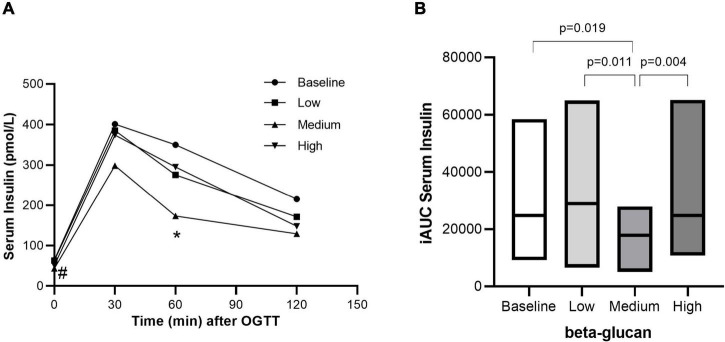
Postprandial serum insulin response after intake of granola with cereal beta-glucan. The postprandial insulin response from 0–120 min **(A)** and the incremental area under the curve **(B)** after an OGTT at baseline and after consuming low, medium and high amount of beta-glucan for three days. Data is given as median (25^th^–75^th^). Significant differences are calculated with Friedman’s Anova followed by Wilcoxon signed rank test. #Fasting insulin after medium and high beta-glucan significant different from baseline. *Insulin response at 60 min after medium beta-glucan significant different from baseline, low and high beta-glucan.

After three days of intervention, the effect of granola meals with different doses of beta-glucan on glycemic response was determined on the fourth day over a 3h period after an OGTT. Glycemic response was improved with granola intervention containing cereal beta-glucan, although not in a dose-response manner. While the fasting glucose level significantly decreased within the high beta-glucan intervention, there were no difference between groups (Figure 3A and Table 4). Fasting insulin levels were significantly reduced after intervention with medium (*P* = 0.004) and high (*P* = 0.006) beta-glucan meals, compared with baseline (Figure 4A and Table 4). The post-prandial blood glucose response (iAUC) was significantly reduced after intervention with the medium beta-glucan meal compared with baseline (*P* = 0.011), low (*P* = 0.016) and high (*P* = 0.019) beta-glucan meals (Figure 3B). Also, the insulin post-prandial response (iAUC) was reduced after intake of medium beta-glucan meal compared with baseline *P* = 0.019), low (*P* = 0.011) and high (*P* = 0.004) beta-glucan meals (Figure 4B). We further examined two indices, HOMA-IR (38) and Matsuda index (39), reflecting insulin resistance and insulin sensitivity, respectively (Table 5). There were no differences in HOMA-IR after intake of the different amounts of beta-glucan. Matsuda index significantly increased after intake of medium (66.8%) and high (29.2%) beta-glucan meals compared with baseline (*P* = 0.016, *P* = 0.041, respectively), indicating improved insulin sensitivity (Table 5).

**TABLE 4:** Fasting blood glucose and insulin values before and after intake of granola with cereal beta-glucan in different amounts.

	Low beta-glucan			Medium beta-glucan			High beta-glucan			*p-value #*
	Before	After	p-value*	Before	After	p-value*	Before	After	p-value*	
Fasting Glucose (mmol/L)	5.1 (4.8–5.7)	5.2 (4.8–5.5)	0.975	5.3 (5.0–5.7)	5.1 (5.0–5.4)	0.343	5.3 (5.0–5.6)	5.1 (4.8–5.4)	0.032	0.204
Fasting Serum Insulin (pmol/L)	64.5 (58.8–83.8)	63 (37.0–84.5)	0.198	55.0 (37.0–65.5)^a^	43.5 (30.0–62.0)^a^	0.153	56.5 (28.5–67.0)^a^	51.0 (36.5–62.5)^a^	1.00	0.002

*Data is given as median (25^th^–75^th^). p-values indicates differences between and within groups calculated by Friedmans Anova and Wilcoxon signed rank test. N = 14.*

*P-values calculated with Wilcoxon signed rank test within groups. ^#^P-values calculated with Friedmans Anova between groups. ^a^significant different from baseline as calculated with Wilcoxon signed rank test (p < 0.05).

**TABLE 5:** Insulin resistance (HOMA-IR) and insulin sensitivity (Matsuda index) after intake of granola with cereal beta-glucan in different amounts.

	Baseline	Low beta-glucan	Medium beta-glucan	High beta-glucan	*p-value^#^*
HOMA-IR	15.3 (12.3–19.4)	14.2 (7.9–20.0)	10.4 (6.5–14.1)	10.8 (7.8–14.1)	0.193
Matsuda index	14.1 (10.1–16.5)	14.5 (11.3–19.7)	21.7 (16.4–29.4)^a^	17.0 (13.2–25.4)^a^	0.004

*Data is given as median (25^th^–75^th^). Significant differences are calculated with Friedman’s Anova followed by Wilcoxon signed rank test. N = 14.*

***#** p-value calculated with Friedman’s Anova. ^a^significant different compared to Baseline (p < 0.05) calculated with Wolcoxon signed rank test. HOMA-IR: Homeostatic Model Assessment for Insulin Resistance.*

### Glucagon-Like Peptide-2 and Peptide YY After Intake of Granola With Cereal Beta-Glucan

**FIGURE 5:**
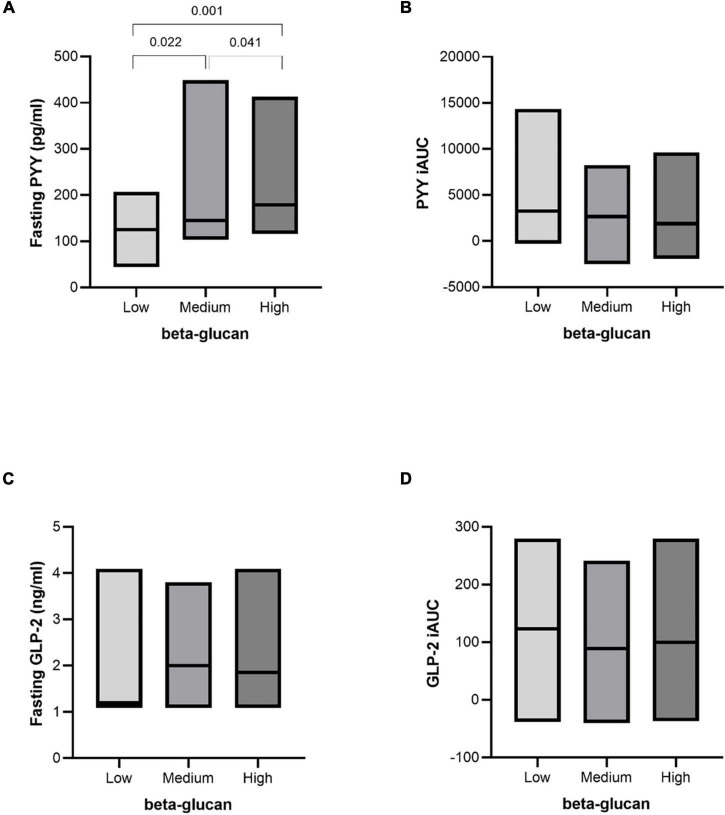
Fasting and postprandial response of PYY and GLP-2 after intake of granola with cereal beta-glucan. Fasting values **(A,C)** and the incremental area under the curve **(B,D)** after an OGTT after consuming low, medium and high amount of beta-glucan for three days. Data is given as median (25^th^–75^th^). Significant differences are calculated with Friedman’s Anova followed by Wilcoxon signed rank test. Significant differences are presented with *p-values.*

Despite a reduction in insulin levels, there were no significant changes in GLP-2 after intervention (fasting and post-prandial) (Figure 5). The fasting level of PYY significantly increased in a dose-responsive manner of beta-glucan content following interventions. Intervention with both medium and high beta-glucan meals increased fasting PYY compared with the low beta-glucan meal (*P* = 0.022 and *P* = 0.001, respectively). In addition, intervention with the high beta-glucan meal increased fasting PYY compared with medium beta-glucan meal (*P* = 0.041) (Figure 5).

### Breath H_2_ and Short Chain Fatty Acids as Markers of Gut Fermentation Activity

**FIGURE 6:**
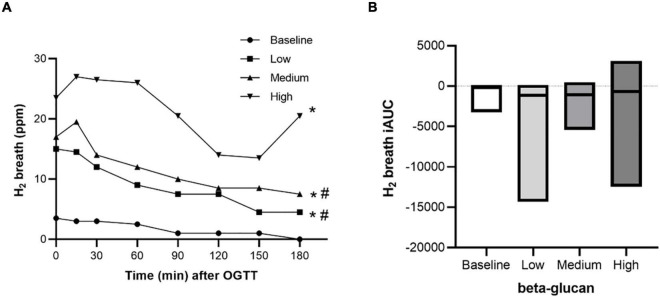
Fasting and postprandial response of H2 breath after intake of granola with cereal beta-glucan. The postprandial response from 0 to 180 min **(A)** and the incremental area under the curve **(B)** after an OGTT at baseline and after consuming low, medium and high amount of beta-glucan for three days. Data is given as median (25^th^–75^th^). Significant differences are calculated with Friedman’s Anova followed by Wilcoxon signed rank test. *H_2_ response (0–180 min) after low, medium, and high beta-glucan significant different from baseline. #H_2_ response (0–180 min) after high beta-glucan significant different from low and medium beta-glucan.

**FIGURE 7:**
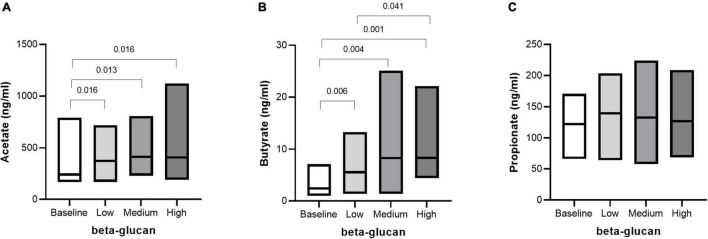
Fasting SCFA after intake of granola with cereal beta-glucan. The fasting levels of Acetate **(A)**, Butyrate **(B)** and Propionate **(C)** at baseline and after consuming low, medium and high amount of beta-glucan for three days. Data is given as median (25^th^–75^th^). Significant differences are calculated with Friedman’s Anova followed by Wilcoxon signed rank test and are presented with *p-values.*

Microbial fermentation activity was measured by monitoring breath H_2_ and SCFA in plasma. A beta-glucan dose-dependent increase in fasting breath H_2_ was shown, but this was not reflected in the post-prandial response (iAUC) after the OGTT (Figures 6A,B). SCFA are end products of gut microbiota fermentation and are absorbed into the circulation. After three days of intervention with beta-glucan, fasting plasma levels of acetate and butyrate increased independently of dose, compared with baseline (Figure 7). In addition, the plasma level of butyrate also increased after intervention with the high beta-glucan meal compared with the low beta-glucan meal (*P* = 0.041). There were no significant changes in propionate (Figure 7). We also investigated the correlation between the glycemia response and the SCFA. Fasting acetate was negatively correlated with glucose response (AUC) (*r* = 0.338, *P* = 0.011). Propionate was positively correlated with fasting insulin (*r* = 0.269, *P* = 0.045) and insulin response (iAUC) (*r* = 0.263, *P* = 0.050) (Data not shown).

### Intervention Effect on the Gut Microbiota

The gut microbiota of the participants was analyzed in feces, and interestingly, all the participants were dominated by Firmicutes (mean abundance of 77%), followed by Bacteroidetes (13%) and Actinobacteria (6%) at baseline. Even though a high inter-individual variability in microbiota composition was found between the participants, explaining ∼70% of the total variation, a significant intervention effect (*P* < 0.001) was also evident. The intervention effect explained ∼6% of the total variation between the visits. There was a dose-dependent discrimination power (per meal vs. representative baseline) of 35%, 43.7%, 56.6% for the low, medium and high beta-glucan meals, respectively. However, the intervention had no significant impact on the microbial diversity (all metrics *P* > 0.1).

**FIGURE 8:**
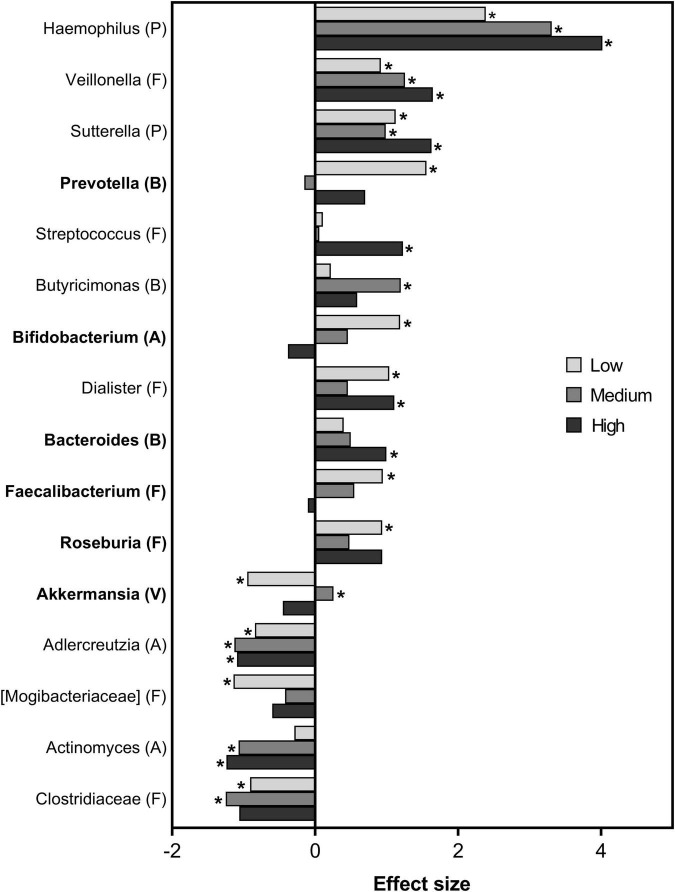
Bacterial taxa after intake of granola with cereal beta-glucan. The taxa order is sorted from high to low effect size independent on test meal. The effect sizes were calculated as the difference in log ratio group means of test meals vs representative baseline after adjusting for individual differences. Asterisk indicates significant relationship (VIP > 1). Bars are colored according to dose of beta-glucan. Dominating genera (average abundance > 1%) are indicated in bold. Phyla are indicated within parentheses, A: Actinobacteria, B: Bacteroidetes, F: Firmicutes, P: Proteobacteria, V: Verrucomicrobia.

Overview of the genera significantly affected by one of the three test meals is presented in Figure 8, shown as effect size per test meal compared to representative baseline. Highest positive effect size was of *Haemophilus*, followed by *Veillonella* and *Sutterella*. This effect was common for all the three test meals, but with largest effect with the high beta-glucan meal. *Streptococcus, Dialister, and Bacteroides* were also positively affected by the high beta-glucan meal. The medium beta-glucan meal was reflected by positive effect size of *Butyricimonas* and a slight effect of *Akkermansia*. Specific for the low beta-glucan meal was positive effect sizes of *Prevotella, Bifidobacterium, Roseburia* and *Faecalibacterium*.

**FIGURE 9:**
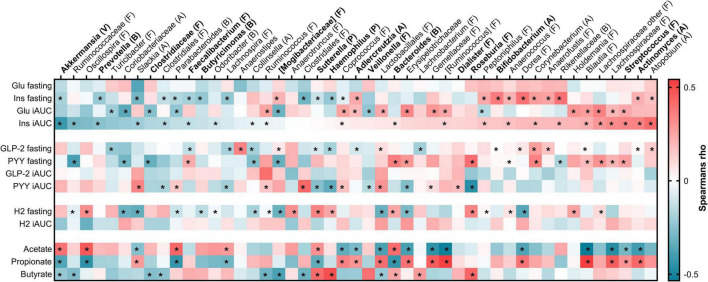
Heatmap of Spearman’s rank correlation coefficient between bacterial taxa and metabolic parameters. The bacterial taxa are sorted from negative (blue) to positive (red) correlation toward insulin iAUC, and those affected by the test meals are indicated in bold. Significant relationship was set to VIP > 1 and indicated by asterisk. No relationship was determined between the bacteria and glucose fasting, GLP-2 iAUC or H2 iAUC. Phyla are indicated within parentheses. A: Actinobacteria; B: Bacteroidetes; F: Firmicutes; Glu: Glucose; GLP-2: Glucagon-like peptide 2; H_2_: Hydrogen; iAUC: Incremental area under the curve; Ins: Insulin; P: Proteobacteria; PYY: Peptide YY; V: Verrucomicrobia.

We further investigated to what extent the observed differences in biomarkers of metabolic regulation were related to the genera (Figure 9). The taxa were sorted according to the correlation with insulin iAUC, the primary end point in the study. Indeed, several of the bacterial taxa that were affected by the beta-glucan meals showed correlation to biomarkers of glycemic response. Of them, highest negative correlation to insulin iAUC was observed for *Akkermansia*, while *Streptococcus* and *Actinomyces* were highly positively correlated to insulin iAUC. Negative correlation to fasting insulin was observed for *Akkermansia*, *Prevotella*, *Sutterella* and *Haemophilus*, and the opposite observed for *Bifidobacterium*.

Positive correlation to fasting PYY was among others observed for *Bacteroides* and *Roseburia*, while the latter was negatively correlated to PYY iAUC as well as *Haemophilus, Veillonella and Sutterella*. The SCFA acetate and butyrate, both affected by the beta-glucan meals, were both positively correlated with *Sutterella* and *Bacteroides*. Acetate was also positively correlated to *Akkermansia*, while butyrate was highest correlated to *Haemophilus* and *Roseburia* (Figure 9).

No relationship was determined between the bacteria and fasting glucose, GLP-2 iAUC or breath H_2_ iAUC (Figure 9).

### Physiochemical Properties of Test Meals

To characterize the physiochemical properties of the different test meals, including viscosity, the test meals were subjected to an *in vitro* digestion (Table 1). Viscosity after digestion was high for the high beta-glucan test meal (114 mPas), reflecting the high beta-glucan dose, molecular weight and relatively good solubility (40% of total beta-glucan was solubilized during simulated digestion). The medium beta-glucan test meal had a lower viscosity (3.2 mPas) due to a lower dose and lower solubility (28%). The low beta-glucan test meal exhibited a viscosity similar to water (1.1 mPas). Under physiological conditions (e.g., *in vitro* digestion), the medium beta-glucan test meal had the highest amount of insoluble, indigestible carbohydrate (20.4 g/100 g), followed by the low beta-glucan test meal (18.2 g/100 g) and the high beta-glucan test meal (17.4 g/100 g). This difference was due to glucose polymers, e.g., insoluble beta-glucan, cellulose and resistant starch, while insoluble arabinoxylans did not differ between test meals (Table 1).

## Discussion

In the present study, we show that intake of cereal beta-glucan as part of a granola meal for only three days improves glycemic response after a glucose challenge, increases blood levels of PYY and SCFA and concomitantly alters the gut microbiota profile in healthy, normo-glycemic individuals. The effect of beta-glucan on glycemic response were not dose-dependent and not related to the ability to form a viscous solution as measured by *in vitro* analyses of the test meals. This is to our knowledge the first study with control on both the dose and the molecular weight of the beta-glucan in relation to glycemic regulation the following day.

### Effect of Granola With Cereal Beta-Glucan and Its Physicochemical Properties on Glycemic Response

The ability of beta-glucan to reduce post-prandial glycemic response is well established (43) and has been ascribed viscosity formation in the intestine which is determined by dose, solubility under physiological conditions, and molecular weight (44, 45). In the present study, both medium and high beta-glucan meals had beta-glucans with high molecular weight, but differences in dose and solubility (higher for high beta-glucan meal) resulted in a huge difference of viscosity after simulated digestion (Table 4). Interestingly, despite the high viscosity of the high beta-glucan meal, the effect on glucose regulation after the OGTT was low, indicating that the effect of beta-glucan on glucose regulation at subsequent meals involves other mechanisms than viscosity.

In support of these results, several studies have shown the beneficial effects of high fiber barley products, on glycemic regulation (18–20, 24, 46, 47). In these studies, a combination of high resistant starch and high dietary fiber content was identified as important for glycemic regulation. However, dose or physicochemical properties of the beta-glucan were not determined. In the present study, we have investigated the impact of cereal-based meals with different beta-glucan doses on glycemic regulation after an overnight fast. Interestingly, only the meal with the medium amount of beta-glucan (3.2 g) significantly reduced both blood glucose and insulin levels after the OGTT. Our results indicate that the beneficial effects of beta-glucan on glycemic regulation the following day are not dependent on viscosity, and can be achieved with lower doses than those set by EFSA for reduction of post-prandial glycemic response, which also have been suggested by others (17).

### Effect of Granola With Cereal Beta-Glucan on Gut Microbiota

Only few randomized controlled trials (RCT) have investigated the effect of fiber on gut microbiota and metabolic regulation in healthy, normal-weight individuals (48). Furthermore, the quality and quantity of the fiber are seldom given. In the present fixed order, crossover study, we found several correlations between bacteria in feces and biomarkers of glycemic regulation and SCFA, indicating an interaction between dietary fiber, gut microbiota and glycemic regulation.

*Haemophilus, Veillonella* and *Sutterella* were increased after all interventions and may represent bacteria that are influenced by soluble fiber in general. Both *Haemophilus* and *Sutterella* were also negatively correlated with fasting insulin, fasting GLP-2 and PYY iAUC, and positively correlated with butyrate. This may indicate a relation between fiber, microbiota, and glycemic regulation. PYY and GLP-2 are important hormones in glycemic regulation (27, 49). Furthermore, we found increased *Bifidobacterium* after the low beta-glucan intervention (which was high in inulin), but not after the medium and high beta-glucan interventions. This is in line with a study showing increase in *Bifidobacterium spp.* after *in vitro* fermentation with inulin but not with oat beta-glucan (50). Others have found a dose-dependent increase in *Bifidobacterium spp.* for several fibers, except for beta-glucan (50). The production of PYY were recently suggested to be strongly regulated by SCFA, evident in human enteroendocrine cells where SCFA increased both PYY secretion and mRNA expression (51). While we found no change in the concentration of GLP-2 after intervention, fasting PYY, but not PYY iAUC, significantly increased after intake of both medium and high beta-glucan meals. In a study by Vitaglione et al., fourteen participants were given 3% beta-glucan in bread, resulting in a 16% higher concentration of PYY AUC compared with control bread (52). Health beneficial effects of beta-glucan may therefore be explained through mechanisms including effects on gastric hormones and peripheral glucose metabolism, which have been proposed (53).

*Bacteroides* constitute a large part of the human gut microbiota, and exerts several beneficial effects on human physiology (54). After intervention with the high beta-glucan meal, we found an increase in *Bacteroides*, which was not significant after the low and medium beta-glucan interventions. A high relative abundance of *Bacteroides* after beta-glucan stimulation (3 g per day) has also been shown by others (55), while a decrease has been shown after inulin (50). Taken together, we cannot conclude from the present study whether the changes in Bacteroides is related to the intake of beta-glucan or other types of fiber present in the meal. Furthermore, how these changes might affect human physiology, needs to be further investigated.

Previous studies have drawn a relationship between colonic fermentation, the generation of SCFA and glycemic regulation (22, 24, 56). In the current study, all three test-meals significantly increased fasting acetate and butyrate levels compared to baseline. The observed difference in glycemic regulation of the low, medium, and high beta-glucan meals cannot be explained by differences in SCFA, as no significant differences between the low and medium and medium and high beta-glucan meals were evident. In a study by Fehlbaum et al., they found that butyrate increased with increasing concentrations of beta-glucan and inulin (50). Hence, the lack of a linear dose-response effect of beta-glucan on the SCFA in the present study might be explained by the fact that beta-glucan was replaced with inulin in the low beta-glucan meal. In a recent review by Ashaolu et al., they show that fermentation of several prebiotics by human colonic microbiota *in vitro* increased the production of acetate and butyrate, while propionate was only increased to a smaller extent (57). This is in line with our results, where only acetate and butyrate increased after the interventions. However, others have shown an increase in propionate in feces after intervention with the prebiotic inulin (58). Of the SCFA, butyrate has obtained attention for its beneficial effects on metabolic regulation of the host (22, 59). Human studies have shown that butyrate producing bacteria, such as *Roseburia*, are less abundant in subjects with T2D (60–62), indicating the need of butyrate producing bacteria for normal glycemic regulation and butyrate supplementation in rodents has been shown to improve insulin sensitivity (63) and glucose homeostasis (64). We found a positive correlation between butyrate and several of the bacteria that changed during the intervention; *Sutterella*, *Haemophilus, Roseburia* and *Bacteroides*. Furthermore, we found a negative correlation between butyrate and *Akkermansia* and *Clostridiaceae*, in addition to *Ruminococcus*. Both *Ruminococcus* and *Roseburia* are known butyrate producing bacteria (65) and these bacteria produce butyrate from beta-glucan and inulin, respectively (50). Taken together, the relation between beta-glucan, butyrate producing bacteria and glycemic regulation needs to be further investigated.

Within the different phyla, the interventions affected the bacteria differently. This was found for example within the Firmicutes phylum where *Roseburia* and *Faecalibacterium* increased only after the low dose, *Dialister* after the low and high dose, *Streptococcus* after the high dose, while *Veillonella* increased after all three doses. This demonstrates the importance of analyzing at a lower taxonomic level (genus/species). The present intervention lasted for only three days, yet it was possible to detect changes in gut microbiota. Others have also demonstrated dietary induced changes in the microbiota in humans after 1–3 days (66). Alterations in gut microbiota by whole grain oats have also been demonstrated by a number of *in vitro* fermentation models (67–70), in addition to animal studies (71–74).

The present results indicate that the high beta-glucan meal with the highly soluble high molecular weight beta-glucans have little effect on glycemic regulation at subsequent meals. Instead, the complex mixture of dietary fibers in the medium beta-glucan meal might have modulated glycemic control, perhaps by modulating the gut microbiota. Other studies indicate that independent of the dose, variation in the chemical structure of a prebiotic can affect its selective fermentation by bacteria (75, 76). In the high beta-glucan meal the beta-glucan were mainly extruded, in contrast to the medium and low dose products dominated by flakes. Others have shown that extrusion increases the solubility of fibers, such as beta-glucan, resulting in increased viscosity of the fiber (77) and hence may affect the gut microbiota differently (78). The presence of extruded beta-glucan in the high test meal could therefore explain the different effect on glycemic response mediated by the medium and the high beta-glucan test meals.

The present study has several strengths. The study had a crossover design, and participants were instructed not to eat oat and barley two weeks prior to and throughout the study. In the present study, we investigated the effect of three different amounts of cereal beta-glucan on glycemic regulation: low, medium, and high doses of beta-glucan corresponding to 0.8 g, 3.2 g and 6.6 g beta-glucan. In contrast to other studies, both beta-glucan quantity and quality (molecular weight, solubility, and viscosity) in the test meals were reported. All of which strengthens the study results. The test meals contain different indigestible carbohydrates (inulin, beta-glucan, other cereal fibers, and resistant starch) that all have the potential to impact glycemic regulation, and hence makes it difficult to draw single ingredient-based conclusion. In addition, the intervention was not designed as a fully controlled dietary intervention and the participants did have other sources of fiber besides the test meals. The participants were instructed to refrain from food rich in beta-glucan throughout the study and we have therefore focused on the effect of the test meals in relation to beta-glucan. Nevertheless, the meals and the amounts of fiber are relevant in a daily diet and shows the impact of indigestible carbohydrates on gut microbiota and glycemic regulation within a short period of time. The small number of participants, the short intervention period, the lack of a 45 min timepoint after the OGTT, not randomized or fully controlled study, and a majority of women are, however, limitations needed to be taken into account when interpreting the data.

To summarize, our results suggest that intake of a granola meal containing 3.2 g of cereal beta-glucan improved glycemic regulation, after only three days in healthy individuals. However, we cannot rule out that also other factors that beta-glucan have influenced the results in the present study. Nevertheless, the results show that it is possible to improve glycemic regulation with doses achievable in a daily diet. The observed effect was not related to viscosity formation in the stomach or small intestine and might instead be related to alterations in the gut microbiota. To fully understand the mechanistic link between beta-glucan intake, gut microbiota and glycemic regulation, more studies are needed.

## Data Availability Statement

The datasets presented in this article are not readily available because of ethical and data privacy restrictions. Requests to access the datasets should be directed to the corresponding author.

## Ethics Statement

The study involving human participants were reviewed and approved by the Regional Committees for Medical and Health Research Ethics (REK 2016/648). The patients/participants provided their written informed consent to participate in this study.

## Author Contributions

VHT-H, SMU, KBH, MGB, and MCWM formulated the research questions and designed the study. VHT-H, LG, BH, and MCWM conducted the intervention study. AR, SB, and SHK performed the dietary fiber analysis and *in vitro* digestion. MGB provided study products. IM and IR performed the microbiota analysis. LG, BH, IM, IR, and MCWM performed statistical analyses. VHT-H, LG, BH, SMU, KBH, IM, SHK, SB, AR, IR, and MCWM interpreted the data. VHT-H drafted the manuscript. VHT-H and MCWM had primary responsibility for the final content. All authors contributed with critical revising of the manuscript and, read and approved the final version of the manuscript.

## Conflict of Interest

The study was performed in collaboration with the food industry (Mills AS) represented by MGB, and Mills AS partially funded the study. VHT-H has been employed at Mills AS. VHT-H does not own any stocks in the company, and the work performed in this manuscript was done after she left the company. VHT-H collaborates with and/or has received research grants from Mills AS, Mesterbakeren, Det Glutenfrie Verksted and Norwegian Celiac Disease Association, none of which are related to the content of this manuscript. SMU has received research grants from TINE, Mills AS, Nortura and Olympic Seafood, none of which are related to the content of this manuscript. KBH has received research grants and/or personal fees from TINE, Mills AS, Amgen, Sanofi and Olympic Seafood, none of which are related to the content of this manuscript. MGB is employed at Mills AS. MGB does not own any stocks in the company and was not involved in the collection or analysis of the data. MCWM is involved in projects with research grants from Tine, Olympic Seafood, Mesterbakeren and Det Glutenfrie Verksted, none of which are related to the content of this manuscript. The remaining authors declare that the research was conducted in the absence of any commercial or financial relationships that could be construed as a potential conflict of interest.

## Publisher’s Note

All claims expressed in this article are solely those of the authors and do not necessarily represent those of their affiliated organizations, or those of the publisher, the editors and the reviewers. Any product that may be evaluated in this article, or claim that may be made by its manufacturer, is not guaranteed or endorsed by the publisher.
